# Flexible and wireless metasurface coils for knee and elbow MRI

**DOI:** 10.1186/s41747-024-00549-8

**Published:** 2025-01-30

**Authors:** Daniel M. Düx, Robert Kowal, Lucas Knull, Simon Schröer, Othmar Belker, Dominik Horstmann, Moritz Gutt, Holger Maune, Oliver Speck, Frank Wacker, Bennet Hensen, Marcel Gutberlet

**Affiliations:** 1 Department of Diagnostic and Interventional Radiology, Hannover Medical School, Hannover, Germany; 2 Research Campus STIMULATE, Otto-von-Guericke University, Magdeburg, Germany; 3 Microwave and Communication Engineering, Otto-von-Guericke University, Magdeburg, Germany; 4 Department Biomedical Magnetic Resonance, Otto-von-Guericke University, Magdeburg, Germany

**Keywords:** Elbow, Knee, Musculoskeletal diseases, Magnetic resonance imaging, Signal-to-noise ratio

## Abstract

**Background:**

Metasurface coils (MCs) are a promising magnetic resonance imaging (MRI) technology. Aiming to evaluate the image quality of MCs for knee and elbow imaging, we compared signal-to-noise ratio (SNRs) obtained in standard clinical setups.

**Methods:**

Knee and elbow MRI routine sequences were applied at 1.5 T, implementing four coil scenarios: (1) 15-channel transmit/receive knee coil; (2) four-channel multipurpose coil (flex coil); (3) MC + spine coil; and (4) MC + multipurpose coil. Three regions of interest (ROIs) at different anatomical depths were compared.

**Results:**

Seven participants (aged 28 ± 2 years; 6 males) were enrolled. In elbow MRI, the MC + spine coil demonstrated the highest SNR across all ROIs (superficial-anterior: +114%, *p* = 0.008; middle: +147%, *p* = 0.008; deep-posterior: +24%, *p* = 0.039) compared to the flex coil and all ROIs, except the deepest from the MC, compared to the knee coil (superficial-anterior: +28%, *p* = 0.016; middle: +104%, *p* = 0.008; deep-posterior: −1%, *p* = 0.531). In knee MRI, the MC + spine coil provided higher SNR compared to the flex coil, except posterior (superficial-anterior: +69%, *p* = 0.008; middle: +288%, *p* = 0.008; deep-posterior: −12%, *p* = 0.148) *versus* the knee coil, the MC + spine coil was superior in the middle but non-different in superficial pre-patellar areas and less in deep-posterior areas (superficial-anterior: −8%, *p* = 0.188; middle: +44%, *p* = 0.008; deep-posterior: −36%, *p* = 0.016).

**Conclusion:**

Wireless MCs exhibited great potential for knee and elbow MRI outperforming the flex coil. Future developments will improve the posterior illumination to increase its clinical value.

**Relevance statement:**

MCs offer enhanced versatility, flexibility, and patient comfort. If universal MC designs can achieve image quality comparable to those of standard coils and simultaneously be utilized across multiple body areas, the technology may revolutionize future musculoskeletal MRIs.

**Key Points:**

- MCs are promising in MRI, but homogeneity is challenging depending on the design.
- Signal-to-noise-ratio was improved for knee and elbow imaging with slight inhomogeneous illumination.
- MCs could match the image quality of standard coils in both knee and elbow imaging.

**Graphical Abstract:**

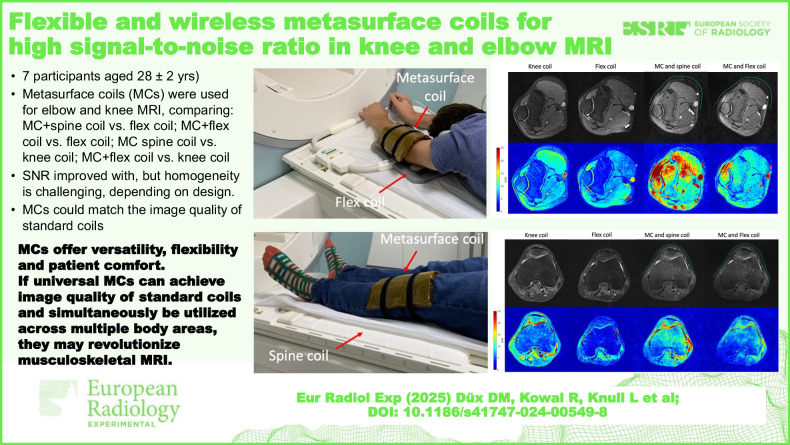

## Background

Musculoskeletal magnetic resonance imaging (MRI) forms an essential part of daily clinical practice. The diagnostic capabilities range from acute trauma, and degenerative/rheumatic diseases to soft tissue and bone tumors, respectively [1]. However, current imaging techniques are limited by highly specialized coil designs tailored to specific anatomical regions. The efficacy and availability of these coils vary across vendors and may add substantial costs for smaller medical centers [2]. Additionally, dedicated coils need to be exchanged between different scan areas, which can lead to a more complicated workflow as opposed to positioning with multipurpose coils.

The introduction of metasurface coils (MCs) offers a promising alternative to conventional MRI coils. MCs contain unit cell resonators, significantly smaller than the radiofrequency wavelength, which can modify the sensitivity of conventional RF coils by inductively coupling inside the scanner [3, 4]. They are known for their ultra-lightweight, flexible designs and the capability to image without cables inside the bore [3], which is relevant not only for patient comfort but also for electromagnetic safety. A common vulnerability in the design of most conventional coils, including transmitting/receiving coils, lies in their cable connections to the scanner. These connectors must be durable to withstand frequent plugging and unplugging, while also ensuring that high-power RF cables do not compromise patient safety or comfort. Wireless MCs may lead to undesirable increases in the specific absorption rate or flip angles. However, this can be mitigated if the MC is designed self-detuning, which makes them receive only MCs. Due to the lack of scanner interfaces, these can be used independently from scanner manufacturers given equal Larmor frequency.

Volumetric MCs have already been investigated for knee and wrist MRI with promising results at 1.5 T and 3 T [5–9]. However, their design is cylindrical to achieve a homogenous signal throughout the image and they are fixed in size, which limits the application to specific anatomical regions for optimal performance. Non-volumetric MCs have shown limited penetration depth in wrist imaging at 1.5-T MRI and have not been further investigated for musculoskeletal applications [10].

Our recent work demonstrated a non-volumetric MC with receiving capabilities only (detuned during RF transmission), whose field homogeneity and penetration depths depended on the shapes, sizes, and arrangements of the unit cells [11]. The optimal setup demonstrated a grid arrangement of the unit cells, which showed better performance with an increasing number of unit cells. The advantage of this configuration compared to previous MCs in musculoskeletal imaging is increased flexibility and patient comfort, which can theoretically be applied to various anatomical regions. This study aims to compare the performance of a non-volumetric MC to standard coil setups in musculoskeletal applications at 1.5-T MRI.

## Methods

Healthy volunteers (≥ 18 years; 1 female; 6 male) gave written informed consent to participate in this prospective study after IRB approval (No. 11019_B0_S_202), who were enrolled in January 2024 (Table 1). Exclusion criteria were contraindications for MRI, acute diseases, and non-compliance. MRI of the knee and elbow were conducted with our standard clinical setup. Scan parameters and positioning are summarized in Table 2.

**Table 1:** Characteristics of the participants

	Mean ± standard deviation	Range
Age	28 ± 2 years	26–32 years
Body mass index	23.8 ± 2.1 kg/m²	21.2–26.6

Maximum diameter (in cm) in anterior-posterior direction		
Elbow	8.8 ± 0.7 cm	7.6–9.4 cm
Knee	12.6 ± 0.5 cm	11.9–13.3 cm

**Table 2:** Scan parameters and positioning for the reference setup

Joint	Sequence and scan parameters	Positioning
Elbow	Proton-density-weighted turbo spin-echo, FS axial TR 2,210 ms, TE 39 ms, FoV 159 × 159 mm^2^, number of slices 18, acquisition time 3:12 min:s, slice thickness 3 mm, spacing 3.3 mm, matrix 384 × 288	Superman
Knee	Proton-density-weighted turbo spin-echo, FS axial TR 2,410 ms, TE 40 ms, FoV 151 × 179 mm^2^, number of slices 34, acquisition time 3:43 min:s, slice thickness 3 mm, spacing 3.3 mm, matrix 448 × 302	Supine

Positioning and scan parameters are shown for elbow and knee imaging. Each scan was performed without any relevant changes in scan parameters or positioning to maintain comparability, especially to generate SNR maps of each setup. For fat suppression, fat saturation was used, which is a frequency-selective excitation pulse centered on the Larmor frequency of fat

*FoV* Field of view, *FS* Fat saturation, *TE* Echo time, *TR* Repetition time

**Fig. 1:**
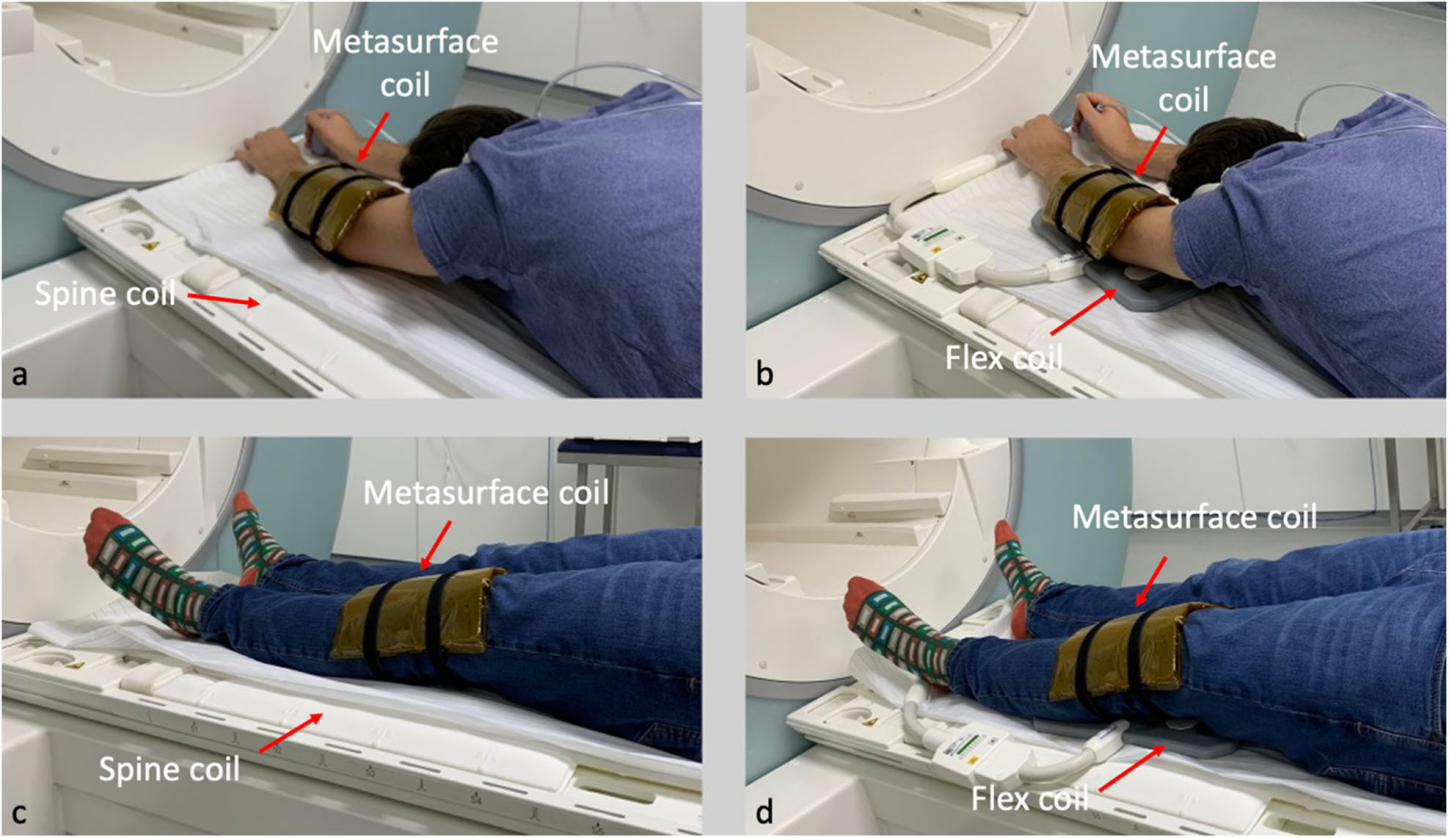
Positioning with the MC (setups 3 and 4). **a** Positioning of the elbow with the MC together with the table-integrated spine coil. **b** Positioning of the elbow with the MC together with the flex coil. **c** Positioning of the knee with the MC together with the table-integrated spine coil. **d** Positioning of the knee with the MC together with the flex coil. The positioning of the knee and elbow was fixated using sandbags and straps, such as used in clinical routine, respectively

All scans were performed on a 1.5-T MRI (Avanto, Siemens Healthineers, Erlangen, Germany). Each scan was performed with four separate setups. Two reference setups represented either the state-of-the-art of a dedicated knee coil, or a multipurpose coil wrapped around the respective joint. The multipurpose coil is usually used if the knee coil is not available or too small in clinical practice. A non-volumetric MC was used in two alternative setups, coupling to either the spine coil or coupling to a separate multipurpose coil (Fig. 1).

The four coil setups were:

1. Transmit/receive 15-channel knee coil (Tx/Rx 15-Channel Knee Coil, Siemens Healthineers) as state-of-the-art setup;
2. Multipurpose receive 4-channel coil (Flex Large 4, Siemens Healthineers) as a typical setup when imaging with a dedicated coil is not possible;
3. MC, receiving with the table-integrated spine coil (Spine 32, Siemens Healthineers);
4. MC, receiving with a multipurpose 4-channel flex coil (Flex Small 4, Siemens Healthineers).

The design of the wireless MC has been described in our previous work [11]. A non-volumetric MC with a size of 19 × 19 cm^2^ and a 16 × 16 grid of resonant unit cells was tuned to the Larmor frequency of 63.6 MHz. The MC was built to enhance the receive signals only and detune itself during transmission using antiparallel limiter diodes [12], which has been demonstrated as a feasible means to detune the circuit. The MC in this study was not constructed to enhance the transmit field. It was embedded in 5-mm thick foam and foil-coated in polyimid. Thermal imaging camera measurements (TiS 10, Fluke, Everett, USA) did not reveal any heating during phantom tests using the same sequences as for the human study (results not shown).

The standard protocol for knee MRI is usually applied with the phase direction in the right–left orientation due to the local transmitting coil. When excited with the body coil with the same field of view as in the standard protocol, aliasing and significant flow artifacts may occur. Therefore, for knee scans with setups 2–4, phase direction anterior-posterior with flow compensation was selected to avoid wrapping artifacts. Other scan parameters were kept identical to allow for direct signal-to-noise (SNR) comparison between all setups. To correct inhomogeneous illumination, the scanner-integrated B_1_ filter was applied in all scans using the MC [13], which applied a homomorphic filter in the post-process. The SNR was still calculated based on non-filtered acquisitions. The setup with the knee coil and flex coil already provided homogeneous illumination, making the B_1_ filter unnecessary. Instead, here the filter introduced noisier anatomical images (results not shown).

**Fig. 2:**
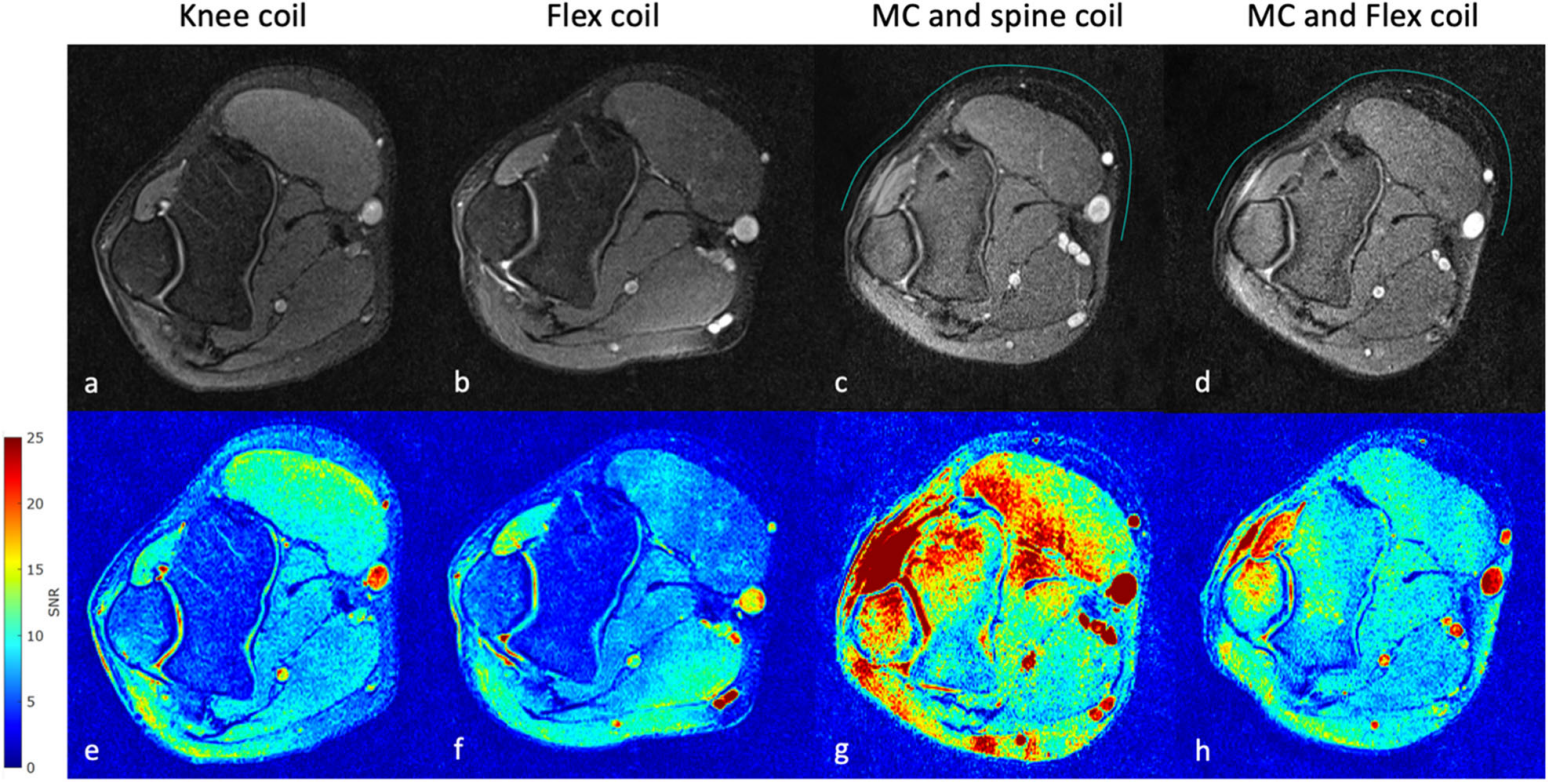
An elbow scan of one participant is shown with the anatomical proton-density-weighted images in the top row and the corresponding SNR maps in the bottom images utilizing different coil setups: knee coil (**a**, **e**); flex coil (**b**, **f**); MC in combination with the spine coil (**c**, **g**); and the MC in combination with the flex coil (**d**, **h**). The green line anterior to the anatomy in (**c**, **d**) illustrates the approximate position of the MC

SNR maps were generated based on noise-only acquisition without radiofrequency transmission for each setup and local noise was calculated as the local standard deviation [14]. Regions of interest (ROIs) A, B, and C were assessed at different anatomical depths, which were consistently evaluable between each MRI acquisition (Figs. 2 and 5). Usually, this location was at the center of the respective coils. For the elbow scan, ROI A at the extensor carpi radialis longus muscle and brachioradialis muscle, ROI B at the trochlea humeri, and ROI C at the pronator teres muscle; For the knee scan, ROI A at the prepatellar soft tissue, ROI B at the femoral bone, and ROI C at the medial head of the gastrocnemius muscle. The SNR of the different coil setups was compared in equivalent ROIs by the Wilcoxon signed rank test. Significance was assumed for *p*-values < 0.05.

MATLAB (version 2023a, The MathWorks Inc., USA) was used for statistical analysis. Data is presented as mean ± standard error of the mean.

## Results

**Fig. 3:**
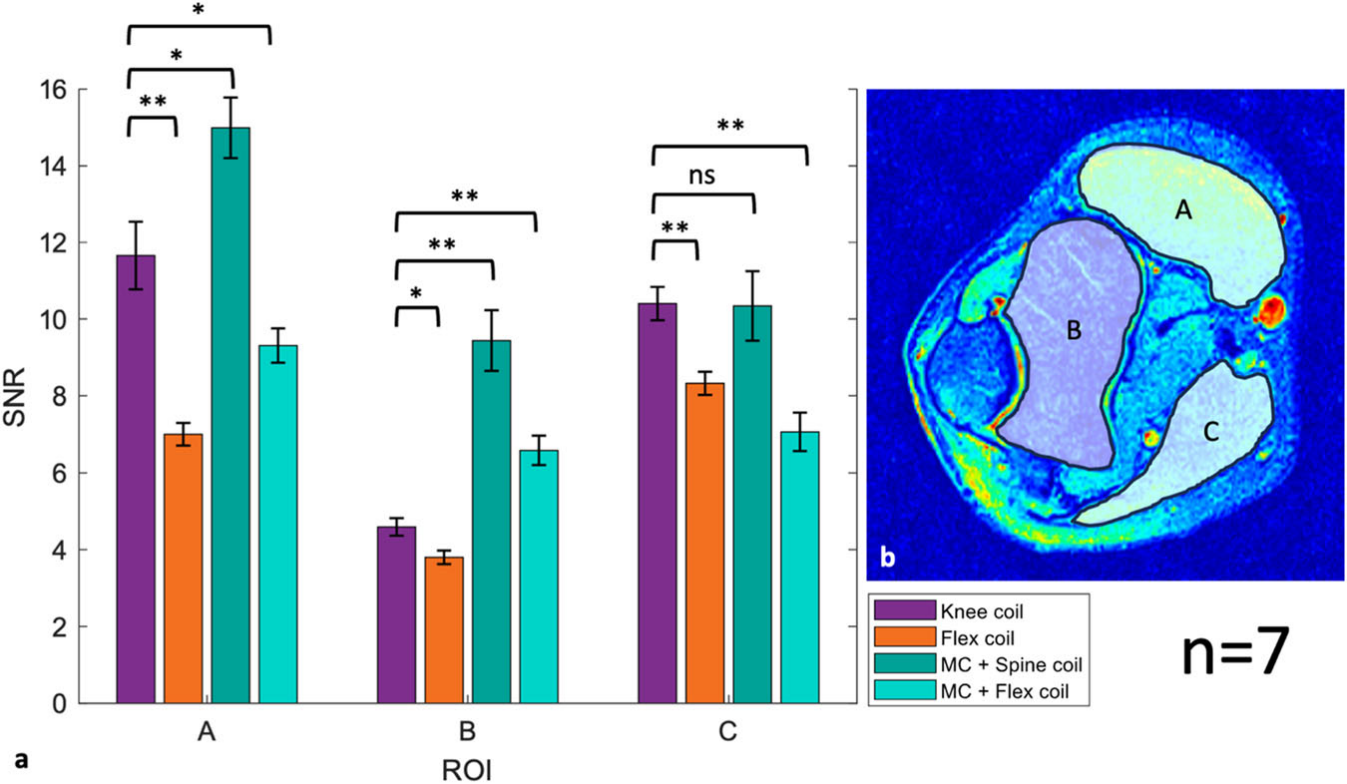
Comparing ROI-based SNR values between each setup in the elbow MRI. The bar chart (**a**) represents signal-to-noise (SNR) values of each set-up based on ROI A, B, and C, which are shown as an example in the image for elbow (**b**). The graph represents the data of the ROIs in all seven participants. Comparing each setup shows that the MC + spine coil setup was superior in ROI A and B, and similar to the knee coil setup in ROI C. MC + spine coil was significantly better than the MC + flex coil. The flex coil setup was the least favorable setup. *p < 0.05, **p < 0.01, MC, Metasurface coil; ns, Not significant; ROI, Region of interest

**Fig. 4:**
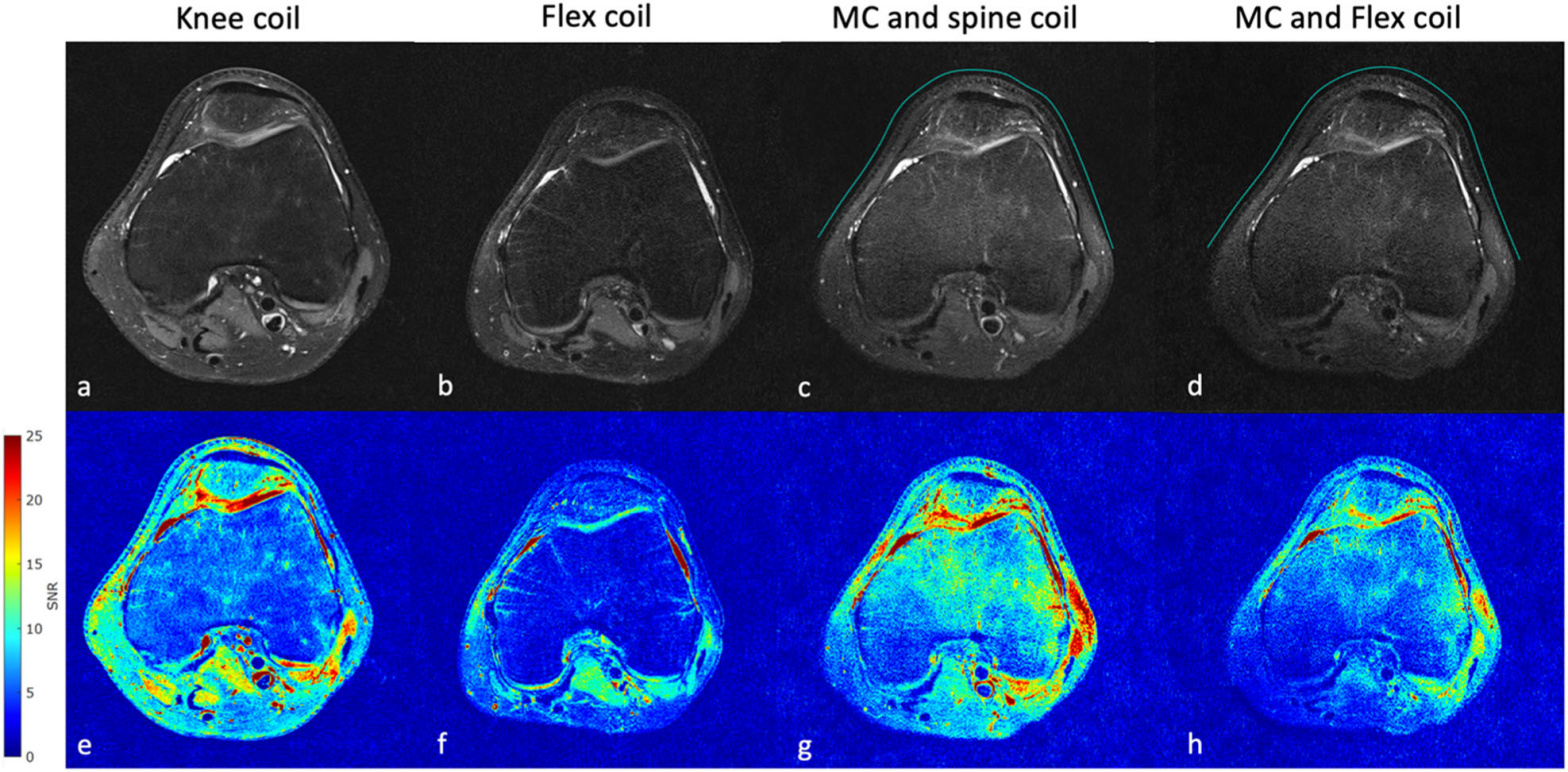
A knee scan of one participant is shown with the anatomical proton-density-weighted images in the top row and the corresponding SNR maps in the bottom images utilizing different coil setups: knee coil (**a**, **e**); flex coil (**b**, **f**); MC in combination with the spine coil (**c**, **g**); and the MC in combination with the flex coil (**d**, **h**). The green line anterior to the anatomy in (**c**, **d**) illustrates the approximate position of the MC

**Fig. 5:**
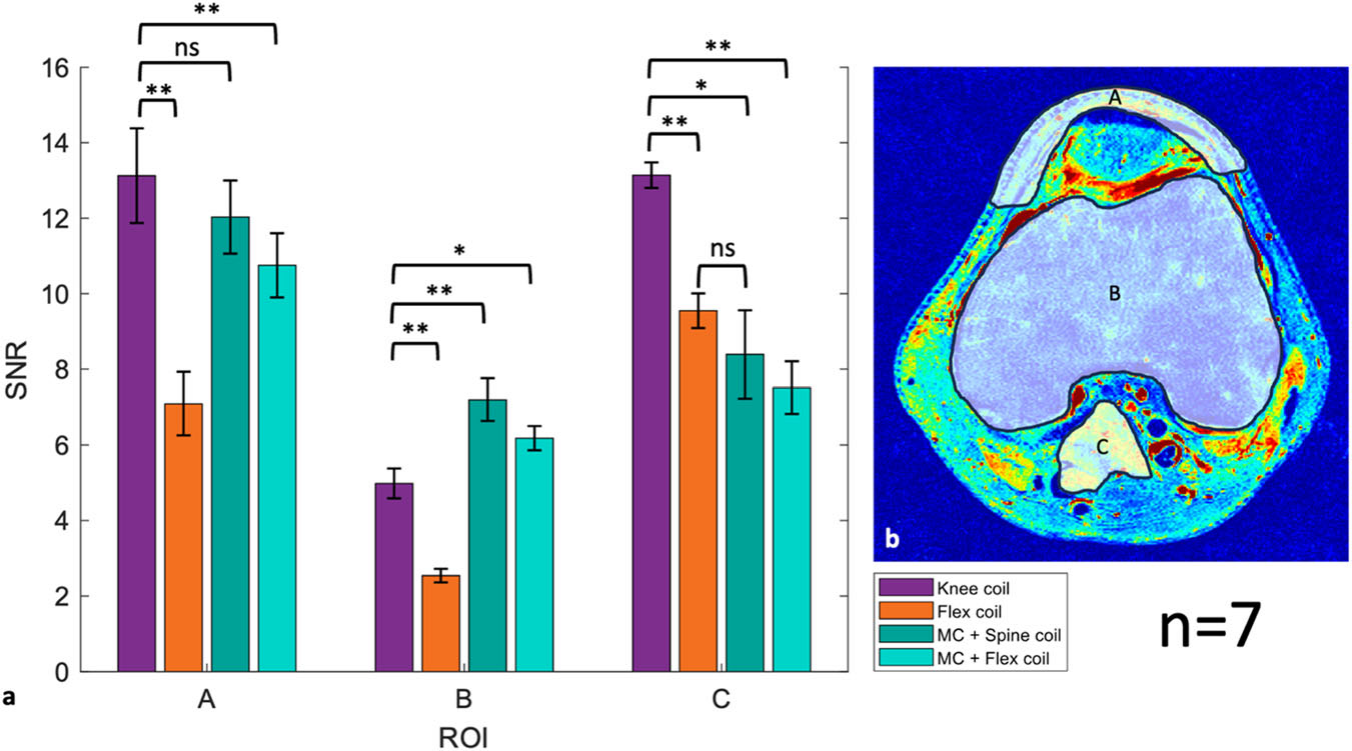
Comparing ROI-based SNR values between each setup in the knee MRI. The bar chart (**a**) represents signal-to-noise (SNR) values of each set-up based on ROI A, B, and C, which are shown as an example in the image for knee (**b**). The graph represents the data of the ROIs in all seven participants. Comparing each setup shows that the MC + spine coil setup was superior in ROI B, similar to the knee coil setup in ROI A, and less favorable in ROI C. In ROI C the setup with the flex coil showed a trend towards better SNR, however, was the least favorable setup in the other two ROIs. MC + spine coil was better than MC + flex coil, however less significant than in the elbow scan. *p < 0.05, **p < 0.01, MC, Metasurface coil; ns, Not significant; ROI, Region of interest

Seven participants aged 28 ± 2 years (mean ± standard deviation), six males, one female) were enrolled. All scans were completed without any restrictions concerning positioning or image acquisition. Imaging examples, corresponding SNR maps, and the ROI assessments are shown in Figs. 2–5. Imaging with the MC resulted in slightly inhomogeneous SNR in both the knee and elbow MRI, as was primarily visible on the SNR maps in areas adjacent to the MC placement. SNR tended to be highest directly below the MC and on its edges where it was fitted to the anatomy, and continuously decreased posteriorly towards the respective coupling coil (spine or flex). After applying the B_1_-filter, anatomical images had improved homogeneity, which was comparable to the state-of-the-art setups. SNR results are shown in Table 3 for elbow and knee imaging; respective ROI assessments are illustrated in Figs. 2 and 5.

**Table 3:** SNR results of different ROIs in elbow and knee imaging

Elbow	Knee coil	Flex coil	MC + spine coil	MC + flex coil
ROI A	11.7 ± 0.9	7.0 ± 0.3	15.0 ± 0.8	9.3 ± 0.4
ROI B	4.6 ± 0.2	3.8 ± 0.2	9.4 ± 0.8	6.6 ± 0.4
ROI C	10.4 ± 0.4	8.3 ± 0.3	10.3 ± 0.9	7.1 ± 0.5

Knee	Knee coil	Flex coil	MC + spine coil	MC + flex coil
ROI A	13.1 ± 1.3	7.1 ± 0.8	12.0 ± 1.0	10.8 ± 0.9
ROI B	5.0 ± 0.4	2.5 ± 0.2	7.2 ± 0.6	6.2 ± 0.3
ROI C	13.1 ± 0.3	9.5 ± 0.5	8.4 ± 1.2	7.5 ± 0.7

SNR values are demonstrated for elbow and knee in all four setups, with the highest value between the setups highlighted. Values are given as means with standard error of mean

*MC* Metasurface coil, *ROI* Region of interest

### MRI of the elbow (Figs. 2 and 3)

ROI A: setup 3 with the MC and spine coil showed the highest SNR and was significantly enhanced compared to the knee coil (*p* = 0.016) and flex coil (*p* = 0.008). Setup 4 with the MC and flex coil was notably less favorable compared to both setups 1 and 3 (*p* = 0.039 and 0.008), respectively. ROI B: significant improvement of the SNR was seen in both setups using the MC compared to the knee coil (*p* = 0.008). The flex coil was the least favorable setup. ROI C: no difference was observed between setups 1 and 3. The SNR in this region was significantly improved with setup 3 compared to setup 2 and 4 (*p* = 0.039 and *p* = 0.008). When utilizing the MC with the spine coil (MC + spine coil), three out of seven participants exhibited slightly lower SNR in soft tissue near the spine coil compared to the knee coil.

### MRI of the knee (Figs. 4 and 5)

ROI A: The setup with the knee coil and MC + spine coil did not show statistically significant differences. However, the setups with the flex coil with/without the MC had significantly less SNR compared to the other two setups (*p* = 0.008). The setup with only the flex coil had almost half of the SNR of the other two setups, whereas the combination with the MC exhibited only slightly less SNR. ROI B: Significant improvement of the SNR was measured in both setups using the MC + spine coil (*p* = 0.008) and the MC + flex coil (*p* = 0.004) compared to the knee coil, respectively. The flex coil was the least favorable setup. ROI C: The knee coil showed significantly higher SNR in this area compared to the flex coil (*p* = 0.008), the MC + spine coil (*p* = 0.016), and the MC + flex coil (*p* = 0.008). The setup with the flex coil by itself showed a trend towards slightly better SNR compared to the setups with the MC, but without significance. Thus, utilizing the MC showed less SNR in the popliteal soft tissue near the respective coupling coil. Pulsation artifacts were minimal in all knee scans.

## Discussion

This study evaluated MRI scans from the elbow and knee to demonstrate the value of non-volumetric flexible MC as an alternative to a dedicated 15-channel transmitting/receiving knee coil or a 4-channel multipurpose coil (flex coil). The knee coil is a state-of-the-art coil in elbow and knee MRI. However, circumstances may arise where this coil cannot be utilized. One such instance is when dealing with obese patients, where the size or design of the knee coil may not accommodate the patient’s body shape adequately. Additionally, anatomical variations or patient discomfort may render the knee coil unsuitable for imaging. Economic considerations can also lead to clinicians improvising with suboptimal setups. In such cases, multipurpose coils such as flex coils serve as an alternative, offering greater flexibility in accommodating diverse patient populations.

Our MC provided significantly improved SNR in elbow MRI for most anatomical regions compared to both the flex coil (ROI B: 147%) and knee coil (ROI B: 104%). In the knee scan, the MC provided improved SNR for most anatomical regions compared to the flex coil (ROI B: 188%) and improved SNR within the internal knee structures compared to the knee coil (ROI B: 44%).

A high SNR is essential for achieving high-resolution MRI exams, which improves the diagnostic sensitivity of pathologic findings. The knee coil is not adapted to elbow scans, which can result in an increased distance of the coil to the anatomy. The MC or the flex coil can be positioned closer to the elbow, which may lead to a more efficient SNR enhancement compared to the knee coil, compensating for fewer channels. Dedicated coil designs aim to fit the coil to the anatomy rather than including many body areas for the most efficient SNR enhancement. Another important factor for coil assessment is the illumination, which was not entirely homogeneous with the MC. Areas distant from the MC did not exhibit significant SNR improvement. However, the scanner-integrated B_1_-filter compensated for this inhomogeneity in the anatomical images. The residual small foci of inhomogeneity near the MC do not seem to be visually disruptive when compared to state-of-the-art setups. Further assessment of the impact of potential misinterpretation of pathological findings (*e.g*., small tears in ligaments or tendons) from inhomogeneity should be further evaluated. Moreover, the signal with the MC appears to be more enhanced in bone compared to soft tissue. This variation in signal impressions could also be due to inhomogeneous illumination. However, this effect is not fully understood yet, and additional investigation is necessary.

When comparing the configurations, that used the MC (either with the spine coil or with the flex coil), the setup with the spine coil yielded superior SNR. However, the SNR difference between both setups was less prominent in the knee scan, suggesting that the MC + spine coil may improve imaging in smaller objects, while the performance of the MC + flex coil remains unclear in larger anatomical regions. The optimal coil combinations regarding the scan region must be further evaluated in future studies. Future work will also address verifying these results on further contrasts and sequence types.

Shchelokova et al [9] evaluated a planar MC for wrist imaging, which seems to lack improvement of the SNR in deep structures of the wrist when coupled to a single-channel coil. Our results show an overall improvement in SNR, with the illumination of deeper structures compared to the MC proposed by Shchelokova et al. However, their MC was designed with a stripe configuration of the resonant cells, which has shown less efficacy than a grid configuration [11]. For our study, a symmetric grid configuration was used, also in a non-volumetric design. The following research with MCs in musculoskeletal exclusively assessed volumetric MCs. These MCs are usually optimized for a specific anatomical region due to their fixed sizes [5–9] and, therefore miss the advantage of being as versatile as non-volumetric MCs.

Additionally, previous MCs for musculoskeletal imaging were often built to not only serve as a receive coil, but also influenced the transmit field. This must be considered by the scanner’s safety measures to limit the specific absorption rate. When comparing a volumetric MC to the flex coil at 1.5 T for wrist imaging in one participant (superman position), the MC resulted in a 136% improvement in SNR for gradient-echo sequences and a 110% improvement for spin-echo sequences within the bone [9]. We achieved comparable improvements in elbow scans regarding SNR. We measured a tendency of stronger improvements in elbow scans, being the smaller anatomy. Wrist imaging should still be evaluated with our MC to prove the validity of this tendency. In 3-T MRI, the same setup achieved an SNR improvement of 81% compared to the flex coil [7]. There is a tendency for less improvement with higher field strengths [11], therefore their results are only indirectly comparable to our scans in 1.5 T.

A similar setup was assessed in 1.5 T for nine participants, comparing a volumetric transmitting/receiving MC to a dedicated transmitting/receiving circularly polarized knee coil [8]. In this comparison, the MC achieved slightly more homogenous and superior SNR compared to the knee coil (*p* < 0.05). The scan time could be reduced by half or alternatively, resolution could be improved while maintaining the same SNR levels between both setups.

Another volume MC was evaluated in eleven volunteers for wrist imaging at 1.5 T [6]. Here three radiologists graded imaging with the MC compared to a flex coil in terms of clarity and artifacts resulting in significantly better image quality as a consequence of significantly superior SNR. This research also showed that image quality was transferable to an MRI of another vendor.

A similar MC setup tested for knee imaging at 1.5 T of United Imaging, Siemens Healthineers, GE Healthcare, and Philips scanner proved that the technology can be transferred across vendors [5]. The MC is usually adapted to one Lamor frequency only, but is compatible with any other vendor with MRIs running at that same Lamor frequency. This is one of the key advantages of MCs, which is potentially very cost-effective because the manufacturing costs of MC are significantly reduced compared to standard coils.

Yi et al [5] conducted knee imaging in ten participants with a 12-channel knee coil, a 4-channel flex coil, an MC + body coil, and an MC + spine coil. The setup MC + spine coil showed better SNR than the MC + body coil, which was also the most favorable MC setup in our study. However, in their study, both tested MC setups resulted in less SNR compared to the knee coil (but superior SNR to the flex coil alone) for *in vivo* scans. The image quality was graded by two radiologists, showing that the MC + spine coil setup appeared significantly better than the knee coil. It was argued that this can be attributed to superior homogeneity when utilizing the MC, which accounts for fewer SNR values.

It is important to recognize that variations in SNR enhancement among these previously mentioned studies may arise from differences in the sizes of the respective MC and reference coils. As noted earlier, volumetric MC exhibits more homogeneous signals [5, 8]. Similar SNR enhancement results might be expected at off-center slices, given comparable dimensions between MC and reference coils, however, future research should validate this.

From an economic standpoint, the MC can have great value for future musculoskeletal imaging. Universal coils such as the proposed flexible MC in this study can be utilized across various body areas, which is more cost-effective compared to the expense of acquiring numerous dedicated coils. This may explain why multipurpose coils are often used as a suboptimal alternative while dedicated coils are not broadly implemented in clinical practice. Not only is this relevant for high-income countries to potentially lower healthcare costs and further increase the availability and quality of MRI. However, MRI also holds a growing role in low- to middle-income countries despite a severe disbalance between availability, maintenance costs, and helium requirements for increasing field strength *versus* the economic capability of the healthcare systems [15]. Additionally, dedicated coils are often not affordable. A solution may be a weaker, more affordable MRI scanner with a versatile and effective coil technique and strong computing power instead of a strong magnet. Our research was done on a 1.5-T MRI, however MCs tend to work better with lower field strengths [11]. This is consequently interesting for low- to middle-income countries, but also for interventional MRI. With better computational power field MRI may potentially increasingly be applied for interventional MRI, because it reduces susceptibility artifacts and increases patient safety while still providing accurate treatment monitoring [16].

This study was limited by a small sample size. Additionally, participants were not obese, which is often common in patients being assessed with MRI in the musculoskeletal domain. An increasing maximum diameter of the anatomy can be challenging when imaging with MC but might become impossible with the knee coil. No parallel imaging was applied in the sequences we used in this study, because it is not part of our usual clinical routine for elbow and knee MRI. It should be noted that parallel imaging with the MC may be challenging due to a reduced number of individual channels.

In conclusion, wireless MCs exhibit great potential for knee and elbow MRI outperforming the flex coil. The optimization of the MC performance represents a multifaceted challenge. While maximizing SNR is essential for signal detection and image clarity, achieving uniform illumination throughout the image is equally critical for diagnostic accuracy. On the other hand, an MC with increased versatility, flexibility, and patient comfort has huge potential. Further research should include larger regions like the hip and shoulder.

## Supplementary information

**Additional file 1: Figure 1:** Imaging with and without applying a B1 Filter for elbow and knee imaging. Figure 2: Evaluation of Effects on B0 of the MC and Flex coil on 3-T MRI. **Figure 3:** SNR maps at the periphery of the Metasurface coil coverage *versus* the corresponding slice of knee and flex coil.

## Data Availability

The datasets used and analyzed during the current study are available from the corresponding author upon reasonable request.

## Abbreviations

MC: Metasurface coil
MRI: Magnetic resonance imaging
ROI: Region of interest
SNR: Signal-to-noise ratio
