# Supplementary material for: Flexible and wireless metasurface coils for knee and elbow MRI
Source: Eur Radiol Exp. 2025 Jan 30;9:13. doi: 10.1186/s41747-024-00549-8 (PMC11782736; doi:10.1186/s41747-024-00549-8)
Supplement: Supplementary file 1 — Additional file 1: Figure 1: Imaging with and without applying a B1 Filter for elbow and knee imaging. Figure 2: Evaluation of Effects on B0 of the MC and Flex coil on 3-T MRI. Figure 3: SNR maps at the periphery of the Metasurface coil coverage versus the corresponding slice of knee and flex coil. [file 41747_2024_549_MOESM1_ESM.pdf]

Flexible and wireless metasurface coils for knee and elbow MRI

ELECTRONIC SUPPLEMENTARY MATERIAL

Figure 1: Imaging with and without applying a B1 Filter for elbow and knee imaging

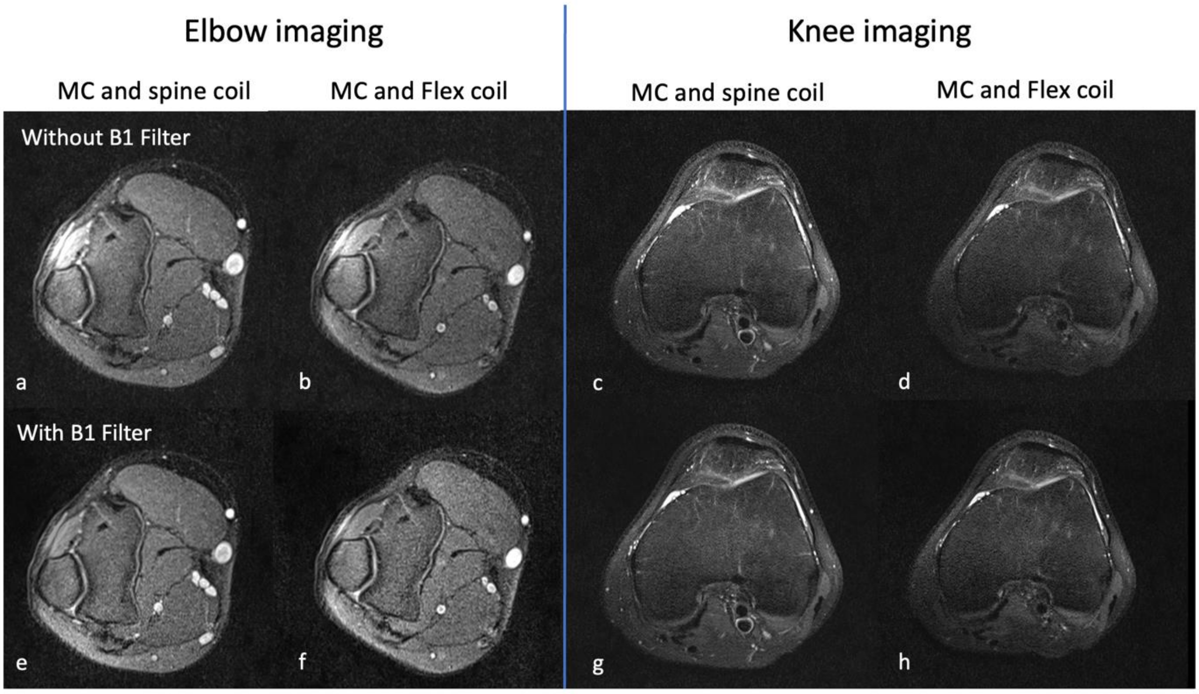

This figure illustrates anatomical images without the B1 filter (A-D) and with the B1 filter (E-H) for the elbow MRI scan (A, B, E, F) and the knee MRI scan (C, D, G, H). SNR maps (shown in the main figures) are based on the imaging without the B1 filter.

Figure 2: Evaluation of Effects on B0 of the MC and Flex coil on 3 T MRI

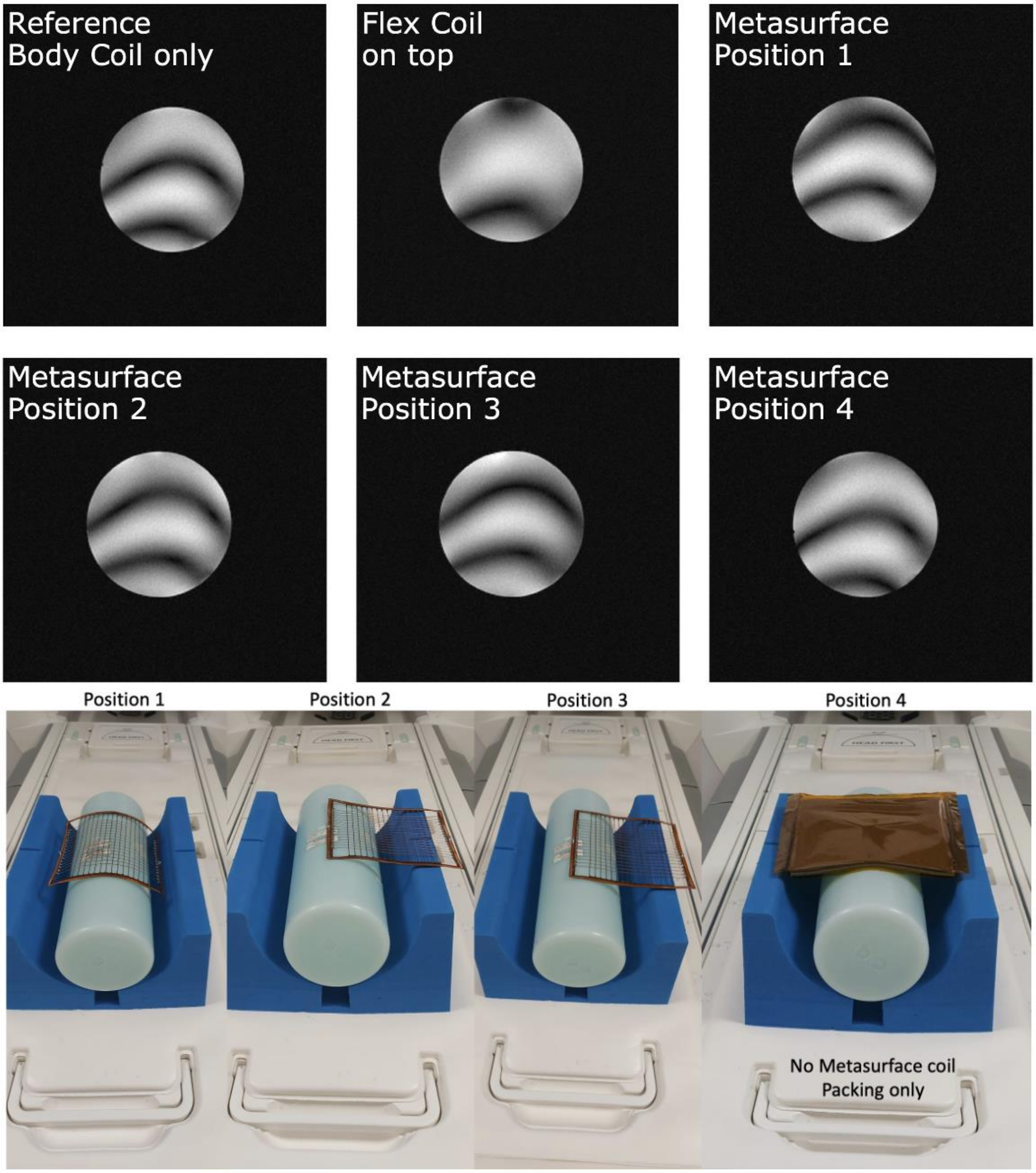

This figure depicts an experiment examining magnitude images with B0 related banding artifacts to showcase minor influence on the B0 field using a vendor implemented protocol, based on a spin-echo sequence with the superposition of regular and stimulated echoes to obtain the homogeneity information. The top two rows display scans including the reference coil (top left), the flex coil on top of the phantom (top middle), and various MC placements on the phantom (labeled as positions 1-3), as well as with just the packing ("Position 4"). Each MC position is illustrated in the bottom row. The MC did not seem to change the B0 field in the respective assessments. The flex coil had minor influence on the B0 field.

Figure 3: SNR maps at the periphery of the Metasurface coil coverage versus corresponding slice of knee and flex coil

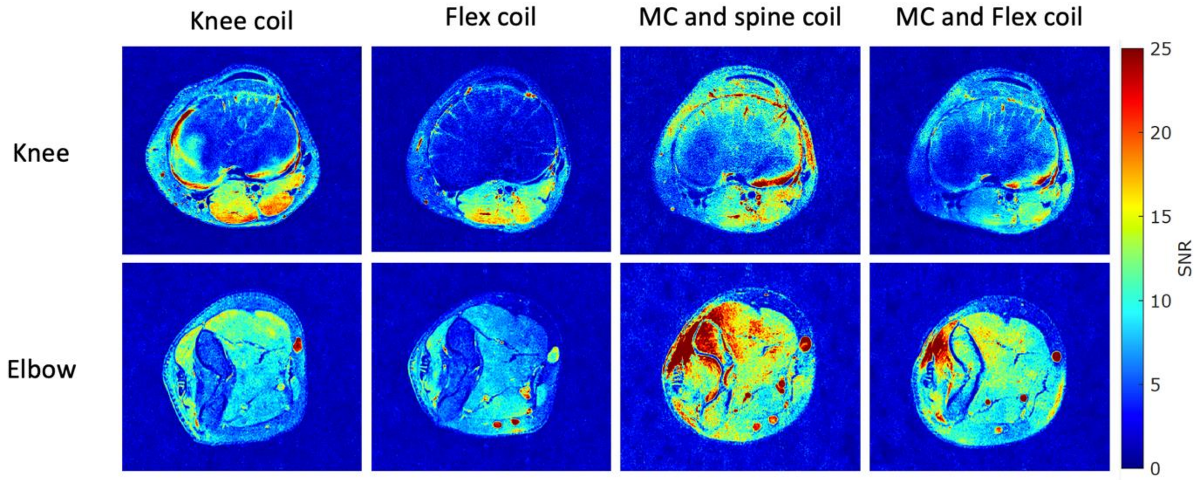

This figure presents knee images (upper row) and elbow images (lower row) using different setups: knee coil, flex coil, MC+spine coil, and MC+flex coil. SNR maps were created at the edge of the MC and compared to the respective slice with the knee and flex coil. Knee imaging is shown 4 cm (12 slices) more cranially and elbow imaging is shown 2 cm (6 slices) more cranially relative to the central slice depicted in the manuscript, respectively.
